# Colombian Stakeholder Perceptions and Recommendations Regarding Fall Detection Systems for Older Adults

**DOI:** 10.3390/geriatrics8030051

**Published:** 2023-05-08

**Authors:** Edna Avella-Rodríguez, Lessby Gómez, Jose Ramirez-Scarpetta, Esteban Rosero

**Affiliations:** 1 Escuela de Ingeniería Eléctrica y Electrónica, Universidad del Valle, Calle 13#100-00, Santiago de Cali 760032, Colombia; jose.ramirez@correounivalle.edu.co (J.R.-S.); esteban.rosero@correounivalle.edu.co (E.R.); 2 Escuela de Rehabilitación Humana, Universidad del Valle, Calle 4b#36-00, Santiago de Cali 760043, Colombia; lessby.gomez@correounivalle.edu.co

**Keywords:** aged, wearable electronic devices, activities of daily living, stakeholder participation, quality of life

## Abstract

This study aimed to analyze perceptions and recommendations from stakeholders on the effectiveness of fall detection systems for older adults, aside from any additional technological solutions they may use within their activities of daily living (ADLs). This study performed a mixed-method approach to explore the views and recommendations of stakeholders concerning the implementation of wearable fall detection systems. Semi-structured online interviews and surveys were conducted on 25 Colombian adults classified into four stakeholder groups: older adults, informal caregivers, healthcare professionals, and researchers. A total of 25 individuals were interviewed or surveyed, comprising 12 females (48%) and 13 males (52%). The four groups cited the importance of wearable fall detection systems in ADLs monitoring of older adults. They did not consider them stigmatizing nor discriminatory but some raised potential privacy issues. The groups also communicated that the apparatus could be small, lightweight, and easy to handle with a help message sent to a relative or caregiver. All stakeholders interviewed perceived assistive technology as potentially useful for opportune healthcare, as well as for promoting independent living for the end user and their family members. For this reason, this study assessed the perceptions and recommendations received concerning fall detectors depending on the needs of stakeholders and the settings in which they are used.

## 1. Introduction

All over the world, populations are aging. By 2050, the population of older adults (aged 60 and over) is projected to exceed two billion [1,2,3]. In Colombia, the National Observatory on Aging and Old Age (ONEV) predicts that by 2035, older adults will comprise 19.09% (10.78% women and 8.31% men) of the total Colombian population [4]. In addition, life expectancy in Colombia has risen over the last 50 years from 62.15 to 77.46, according to The World Bank [5], a direct consequence of better access to health systems and more effective treatments. In Colombia, it is important to note that the majority of the nation’s elderly are non-institutionalized and cohabit with children and/or family members. The remainder (29.2%) live in two-person households, but a not insignificant number (14.4%) live alone [6,7,8]. Keeping up activities of daily living (ADLs) and following a healthy lifestyle are both key for healthy aging, as they help ensure a high quality of life and independent living [9,10]. However, unplanned events such as falls cause minor to severe injuries or even death in older adults, as is evidenced by the World Health Organization (WHO). Falls have also become a public health problem due to the associated mental, physical, and social health repercussions in older adults [11,12]. In the Americas, in 2019, falls were responsible for 1.48% of all deaths of adults over 60 years of age and, in Colombia, they were the cause of 0.75% of deaths, according to a study by the Pan American Health Organization [13].

As life expectancy increases, the healthcare system must invest in more specialized healthcare equipment to provide better coverage and more timely care to older adults. Policies in Colombia regarding the Internet of Things (IoT) are more focused on Internet coverage and use, for example, cell phone calls and messaging [6,14]. The public health system also has low-level use of devices in healthcare employing the Internet of Medical Things (IoMT) [15]. One possible timely and quality care solution could be to use intelligent systems that support older adults in their ADLs and respond to emergency situations, as outlined in the report by the WHO [16]. Various approaches using IoMT assess risk, prevent, and detect falls in older adults [17,18]. One integral approach is reliable fall detection because older adults can receive emergency help rapidly, preventing them from suffering more severe complications such as dehydration and hypothermia, and reducing the mortality rate from falls [19,20]. The growth of IoMT wearable technology for healthcare monitoring has great potential [21], although many older adults ignore the existence of these technologies [22,23,24]. However, the usage of these systems is encouraged, as they can assist in maintaining the autonomy and lifestyle of the elderly by providing timely help. Although some systems may still lack accuracy, the literature has recommended technological solutions with alternative methods for improved detection [25,26,27]. Fall detection systems (FDSs) can usually be classified into two categories.

- User-activated or personal emergency response systems (PERS) are FDSs featuring an alert button which the user can activate manually. Upon activation, a text message is sent or an alert call is made to a specific caregiver, providing them with the user’s geopositioning [28].
- Automatic FDSs detect a fall without requiring activation by the user, while having the advantages of the previous system [28].

Indeed, FDSs employ wearable technology as a frequent solution due to its affordability, tracking capabilities, and compactness, mostly called wearable fall detection system (WFDS) [28]. Some older adults are aware of FDSs but present barriers in accepting such devices due to a perceived difficulty in their use, and a lack of understanding of their purpose and potential benefits. Other older adults, however, are interested in wearing this type of system to feel protected and prevent complications in case of a fall [22,29,30,31]. For example, the analysis made by Camp et al. [22] demonstrates that monitoring ADLs using wearable technology is acceptable, but stakeholders want systems that are user-friendly and which do not employ multiple sensors. The analysis presented by Abdul Rahman et al. [29] indicates that older adults in Malaysia would like to use FDSs, but with training and support for the system wearer. The research conducted by Thilo et al. [32] presents the perceptions of FDSs, considering family members and health professionals. This study shows that family members are more receptive to their parents using this type of technology; however, conflicts arise between users and family members because users can feel monitored or controlled and may not share the concerns of their caregivers regarding their own safety. Another stakeholder group is health professionals who act as mediators in facilitating the decision to use such technology. Although a range of wearable devices are already available, marketed as appropriate for the elderly, some researchers have concluded that further research is required into the technology to ensure it better meets their specific needs [24,33,34].

FDSs aim to provide timely help when a fall occurs by sending timely information to caregivers. Furthermore, in turn, caregivers can go to the assistance of older adults in the event of an injury. Another aspect is that the use of such systems by the elderly should not become mandatory because, while they provide support in emergencies, they do subject the wearer to continuous monitoring. However, data from the monitoring are only shared with their relatives or caregivers in the case of a suspected fall. Therefore, the suggestions, perceptions, and opinions of the multiple stakeholders in FDSs all contribute to a comprehensive examination of how the technology could be improved to suit the needs of older adults.

This study aimed to analyze the perceptions of older adults (≥65 years), informal caregivers, healthcare professionals, and researchers related to FDSs and their use in older adult care.

## 2. Methods

This study conducted a mixed-method approach to explore the perceptions and recommendations of stakeholders regarding the implementation of wearable fall detection systems (WFDSs). The research team consisted of four members. All members had prior experience with research on exoskeleton, gait analysis, and healthcare. One of the researchers (L.G.) had participated in and published qualitative studies. During the interviews, this work used simple and accessible language for all participants. The first question served as an introduction for participants to become familiar with the language and to feel comfortable with the interviewer or survey. Then, this study conducted a series of general questions about the daily routine, experiences associated with falls, knowledge of fall detection solutions, and perspectives on current problems and future possibilities of wearable fall detectors. At the end of each interview or survey, this study asked participants whether they wanted to add anything else, allowing participants to raise issues that the research had not mentioned in the questionnaire. This work adapted the reporting methods and results from the Consolidated Criteria for Reporting Qualitative Research (COREQ). The COREQ checklist was developed to promote the explicit and comprehensive reporting of interviews and focus groups [35,36].

The research team developed and refined the guide for the semi-structured interviews and surveys to be conducted with the focus groups related to participants’ knowledge around falls and the acceptance of WFDSs. Factors such as FDS experience, expertise, perceived benefits, advantages, approval, perceived disadvantages, and the final decision all help to explain the perception of and recommendations in the use of wearable fall detector technology.

Data from participants in this research were obtained through (1) online surveys and (2) interviews. Online surveys are questionnaires composed of a set of open or closed questions using web platforms; this study used the Google Forms platform. Interviews refer to having a one-on-one conversation, either online or in person. In this research, the video call platform Zoom and WhatsApp were used for online interviews. The key difference between these data collection modes was the level of experience or familiarity required for the use of IoT. For health professionals and researchers, the IoT includes tools they often use in their daily lives, but some older adults and informal caregivers have difficulty handling online surveys, making it more convenient and comfortable for those participants to have a conversation.

### 2.1. Setting and Participants

This study included adults between the ages of 18 and 64 and older adults aged 65 years and over. All participants were residents of Colombia and had no known medical conditions. Online surveys were conducted with all health professionals and researchers (14/25), while interviews were conducted with informal caregivers and older adults, online for 6/25 and in person for 5/25. The question for each stakeholder group were divided into 19 for the health professionals, 14 for the researchers, 13 for the older adults, and 15 for the informal caregivers.

### 2.2. Participant Recruitment

This study recruited participants via consultations on web pages, email, local community contacts, and family members. Inclusion criteria were age-related, with older adult participants required to be aged ≥65 years, and all the other participants aged 18 years or older.

**Figure 1:**
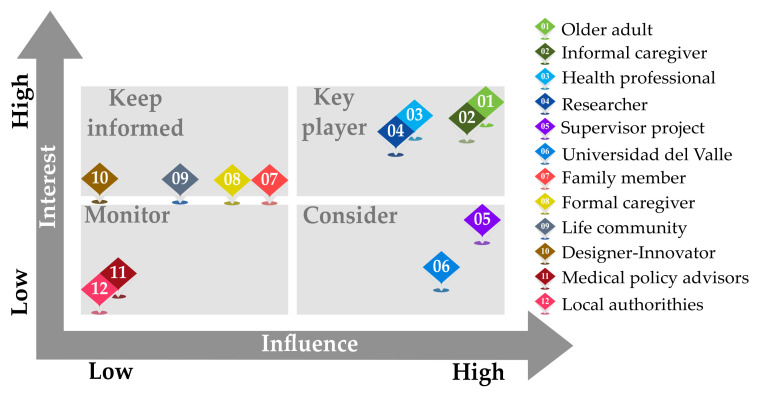
Influence-interest matrix.

The inclusion criteria of stakeholders were according to their interest in and influence on older adult falls. For example, individuals, organizations, or communities that have direct contact with older adults and are involved in their healthcare. This study identified, characterized, and completed an interest-influence matrix in the process of stakeholder selection, as shown in Figure 1. Results show that, evidently, older adults play a key role as system end-users. Another key role is that of the informal caregiver because they have knowledge gained over years of caring for relatives. Healthcare professionals are also an essential piece due to their experience and knowledge of older adult care. Researchers, too, are required stakeholders due to their expertise and scholarship in the intersecting field of health and technology. Other stakeholders are not included in this study because they have medium-to-low influence and interest, as shown in Figure 1. Participants were then divided into four groups: older adults, informal caregivers, healthcare professionals, and researchers with the data gathered through interviews and surveys collated within these four groups.

### 2.3. Ethics Approval

This study was approved by the Health Ethics Research Committee (CEIS), Universidad del Valle, Cali, Colombia (Internal Code 019-021). This study complies with the ethical standards of the Helsinki Declaration and, in Colombia, it complies with Ministry of Health resolution number: 008430 (1993). In addition, the study is considered “research without risk” as it employs documentary research methods such as interviews and online surveys. However, breaches in confidentiality were defined as a potential risk. To this end, the databases have been made confidential and will be used securely, operated only by the project researchers. This study presented informed consent agreements to be signed or accepted by all stakeholders before initiating surveys or interviews. Concerning data from stakeholders, the authors maintained anonymity, using identifiers comprising prefixes and suffixes. For example, this study described the first participant in all categories as an older adult (OA01), informal caregiver (IC01), healthcare professional (HP01), and researcher (RE01).

### 2.4. Data Collection Procedure

This study was conducted between August 2021 and October 2022. Stakeholders who had expressed interest in participating were contacted via email or personal contact. In the surveys, the Google Form link was sent via email. In the in-person interviews, stakeholders were given time to reflect on the information provided and decide whether to proceed. Stakeholders who participated via video call determined the call timing and preferred software for the video call. Throughout the interviews, the interviewer ensured the stakeholder understood the survey information and asked whether the participant wished to continue. Participants confirmed their commitment to the process and gave their consent. During the interviews, the interviewer followed a semi-structured interview guide and provided visuals of the FDS technology. For purposes of analysis, all interviews were audio-recorded.

### 2.5. Data Collection Instruments

#### 2.5.1. Overview

The research team developed the surveys and interview guides. Both the surveys and interviews use open-ended questions, but the surveys also include multiple-choice questions. The decision to use open questions in the design was to encourage participants to share their expectations, experiences, or knowledge of falls and wearable fall detection systems in more detail. During the interviews, the interviewer may need to reformulate questions or clarify concepts or terms so that the participants may internalize the aim and implication of the question, supporting them to respond appropriately. The research divided the interviews and surveys into two categories: fall characteristics and wearable fall detection technology. All surveys and interviews were conducted in Spanish.

#### 2.5.2. Fall Characteristics

The topic of fall characteristics comprised questions to assess fall knowledge and understanding. In the surveys, questions focused on the clinical and research experience of falls, emphasizing assessments, patient opinions, and trends in research. In the interviews, the questions concentrated on the fall incidents of older adults, highlighting the form, quantity, fear, and ADLs as perceived by older adults and informal caregivers. Stakeholders were also asked whether they had any further comments that had not been covered in the survey or interview which could complement the research.

#### 2.5.3. Wearable Fall Detection Technology

This study addresses wearable technology topics in both surveys and interviews, and has components focused on the 4 As—Availability, Accessibility, Appropriateness, and Affordability, besides usability [16,37]. These components are not focusing only on innovation but also on implementing devices that respond to users in the most effective way possible.

### 2.6. Data Analysis

This study analyzed a total of 384 question responses, consisting of 153 responses from interview questions, and 231 responses from the online surveys. Survey responses were subdivided into 98 for researchers and 133 for health professionals. All interviews were audio-recorded and totaled approximately two hours and 51 min, of which 74 min were from interviews with informal caregivers, and 97 min from older adults. Interview responses were transcribed from the audios while survey responses were collated from the Google Forms web platform. Thematic analysis was selected as the approach for interpreting the data because it allows for the categorizing of information as experiences, meanings, views, and opinions derived from surveys, interviews, or conversations [22,38,39]. This study conducted interview and survey questions with the aim of bridging stakeholder and academic perceptions, gathering data on both personal experiences and known fall theories. Indeed, to ensure that this was conducted adequately and uniformly, this study analyzed the questions, coded them, and grouped them into themes. In adopting this thematic coding approach, the research results provide a detailed consideration of stakeholder insights with quantitative data. Subsequently, the data obtained should be saved in a suitable format (Microsoft Excel) to allow for consultation at a later stage.

## 3. Results

### 3.1. Samples and Sociodemographic Data

This study included 25 adults from Colombia, comprising six (24%) older adults (n = 2, 33.33%, women and n = 4, 66.67%, men) aged 65 years and over, and 19 (76%) adults (n = 10, 52.63%, women and n = 9, 47.37%, men) aged 25 to 60 years. From the aforementioned study population, the educational level of surveyed stakeholders (n = 14) is highly educated, the vast majority holding a postgraduate degree (92.86%), and the remainder being graduate professionals (7.14%), while the educational level of the interviewed stakeholders (n = 11) ranges from high school (9.09%), to graduate professional (36.36%), and postgraduate (54.55%). Survey participants were based in Bogotá (n = 4) 16.67%, Popayán (n = 1) 4%, Medellín (n = 2) 8%, and Tunja (n = 1) 4%, while the interviews were conducted in Cali (n = 13) 52%, Firavitoba (n = 2) 8% and Tuluá (n = 1) 4%, overwhelmingly in urban areas (n = 23) 92%, with those in rural areas (n = 2) comprising just 8%.

### 3.2. Findings

#### 3.2.1. Overview

This research divided the results into two main sections: *fall characteristics* and *wearable fall detection technology*, as shown in Table 1. The first section includes (1) frequency or fear of falling; (2) factors that influence falls, such as movements during a fall, recovery movements, or fall type; (3) ADLs in which some older adults, in situations where they need additional help or comfort, request support from their caregivers or family members; and (4) factors that influence the assessment of and research into falls. In the second section, this study separated the *wearable fall detection technology* section into (1) knowledge of FDSs; (2) factors that respond to the needs of stakeholders, ensuring the most effective use possible and maximizing end-user help or support. In addition to the above classification, the items are further categorized into factors of knowledge, incidents, accessibility, affordability, appropriateness, availability, advantages, disadvantages, and use intention. The stakeholders in the survey modality have negative opinions of these systems regarding their reliability, response speed, and domestic market coverage. However, other stakeholders in this same modality highlighted the ongoing importance of fall monitoring in the crucial moment when the user is experiencing a fall. The participants in the interview modality had positive responses about these systems, mentioning the perceived benefits such devices can bring in helping older adults in severe fall cases or other emergencies, but also expressing negative opinions on the issue of unnecessary calls with minor falls. The data stored in the Microsoft Excel spreadsheet provide examples for each section.

#### 3.2.2. Findings for Fall Characteristics

##### Fall Frequency or Fear of Falling

In general, the five informal caregivers reported that the older adults in their care had fallen at least twice in the last ten years and that the falls had occurred when no one was at home. Furthermore, five out of six adults reported having a fear of falls, although the falls they had experienced during the last five years had not led to any serious consequences. However, they expressed fear that, in the next few years, a fall may result in severe bodily injuries which may require emergency help, as well as the fear of not being able to receive timely assistance.

##### Factors That Influence Falls, Such as Fall Type, Movements during a Fall, or Recovery Movements

Overall, of the 18 participants, the most common fall type reported was the forward movement fall (12/18). The healthcare professionals reported that the falls they see most frequently are forward (4/7), followed by sideways, with the backward and downward falls equal in value. Indeed, the older adults and informal caregivers also consider the forward fall the most common, as described by eight (5/6 OA; and 3/5 IC) participants. However, the older adults and informal caregivers consider that the second most common is a backward fall, with sideways and downward mentioned with equal frequency. In the same way, the fall cause most commonly reported is a fall from their own height, followed by slipping while walking and between sitting down/standing up. However, one healthcare professional expressed that


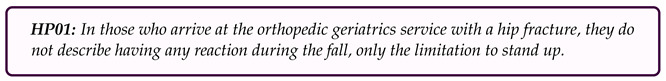


Another aspect is the environment; for example, poor lighting that decreases the visual field, as described by OA04, the false step reported by OA06, and slipping on oil, as described by IC02:


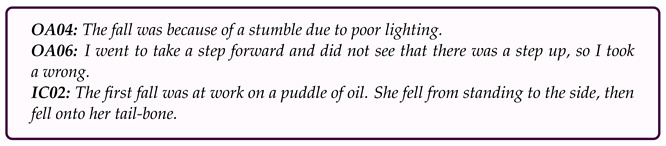


In general, the participants mentioned that the first reaction to a fall is to move their hands to mitigate the force of the fall or to grip something to stop themselves from falling. Similarly, some participants describe that another way to prevent a fall is to take a step, as mentioned by HP05:


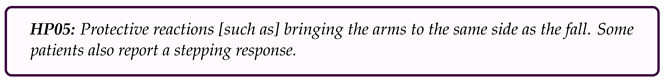


The healthcare professionals mentioned how age and gender may influence falls in older adults. The participants reported that in the age range from 65 to 85 years old, women have more falls than men.

Of the seven healthcare professionals, HP01 refers to an international program of multi-component physical exercises to reduce frailty and fall risk to maintain or increase the independence of older adults. The other survey participants recommended movements that involve motor control exercises, lower limb and trunk strengthening, gait re-education, and workouts to improve proprioception and postural stability. In addition, the healthcare professionals expressed that every intervention must be conducted with security measures for participants.


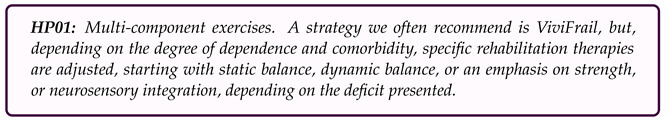


##### ADLs, in Which Some Older Adults Need Help or Comfort, and Request Support from Their Caregivers or Family Members

Generally, older people perform ADLs during the day to care for themselves or others. Likewise, methods or scales are available for the functional assessment of ADLs, from simple to complex activities. This study selected the Groningen activity restriction scale (GARS) to categorize the activities with which older adults require support [40]. Most informal caregivers reported that senior citizens under their care are independent, and their work is complementary, with tasks or duties characterized as heavy activities (IC02-IC05). However, participant IC01 explains that most of the time, he performs extensive and demanding tasks:


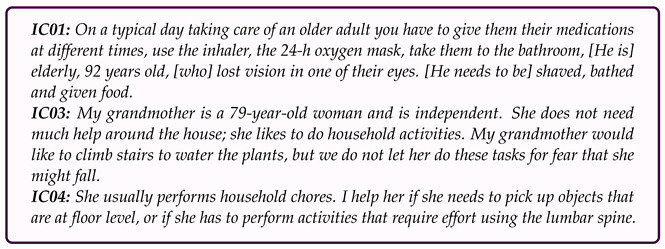


##### Factors That Influence the Assessment of and Research into Falls

Based on the survey results, healthcare professionals perform an average of ten fall risk assessments per month. In fall risk assessment consultation, professionals examine an individual’s mental status, sensory integrity, aerobic capacity, fall history, and medication use in addition to checking cardiovascular/pulmonary, musculoskeletal, and neuromuscular health. These checks are also used alongside other tests, such as mobility, functionality, posturography, and gait patterns. They usually perform further specialist tests to determine fall risk with scales such as Morse, TUGO, SPPB, Dawton, SARC-F Sarcopenia, Frailty Scale, and Barthel. The findings suggest that in the age range of 65–85, women report more falls than men, but aged >86, men fall more. Finally, the professionals carry out interdisciplinary committees to determine whether the patient requires orthopedic support or other types of help.

The surveyed researchers focused on fall topics such as assistive technologies, physical activity and health, biomedical signal processing, artificial intelligence, robotics, bioethical issues, and community with an emphasis on instrumentation, inertial signal processing, fall risk assessment, and biomechanical analysis. In Colombia, research into the technology used in elderly healthcare has been ongoing for approximately ten years, as evidenced by the study developed by Vesga Ferreira et al. [14] identifying trends, regulations, and guidelines for the Smart m-health systems design for biological signals monitoring under an IoT scheme. The system developed by Capera-Peña and Huertas-Prieto [41] implements a prototype with an inertial sensor, detecting some falls in older adults and transmitting data to an IoT platform. The work by Munoz Garcia [15] shows the digital divide of older adults in Colombia from an economic, social, and cultural perspective.

#### 3.2.3. Monitoring Technology Findings

##### Knowledge of Fall Detection Systems

**Figure 2:**
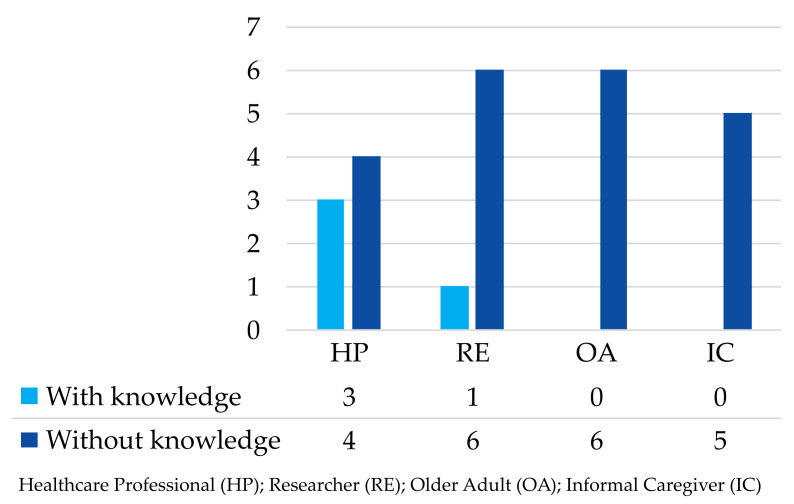
Summary of existing knowledge of WFDSs.

Of the seven healthcare professionals, only three acknowledged the existence of FDSs and just one of those reported knowing about FDSs to improve balance. The remaining four HPs were not aware of this type of system, as shown in Figure 2. The researcher participants reported knowing about FDSs and believe that fall detection employing instrumentation such as inertial or electromyography sensors, phones, or bands may be beneficial in fall assessment, prevention, and detection. The response from the informal caregivers and older adults is that they are unaware of FDSs. In fact, informal caregivers typically described the functions of FDSs based on their conjectures upon hearing the device name.

Some participants talked about research trends in FDS technology: (1) to employ instrumentation as inertial sensors and electromyographs; (2) new algorithm development using fall cause and environments.


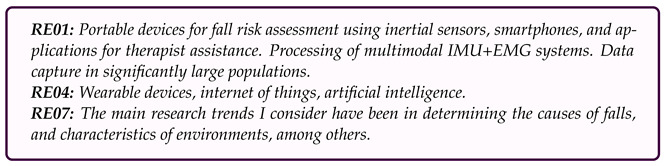


##### Elements That Respond to the Needs of Stakeholders

Of the 25 participants, 21 stakeholders either did not answer or responded in the negative about the ease in which individuals could obtain and adequately use wearable fall detection technologies, with key obstacles being that the stakeholders were unaware of the existence of FDSs or had not had first-hand experience of them. The positive answers about accessibility also include disadvantages due to the issue of false positives or lack of information in experimental validation:


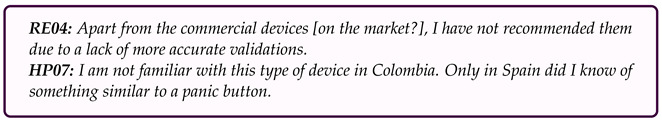


**Figure 3:**
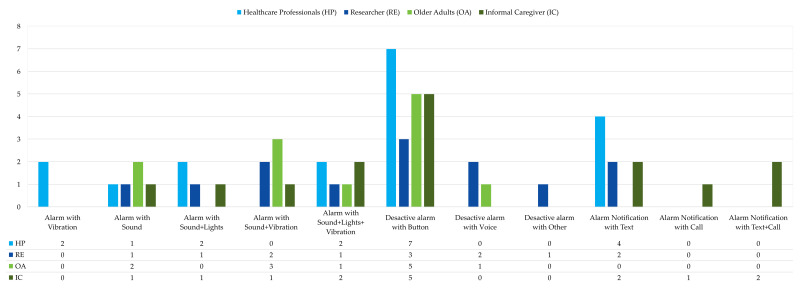
Summary of considerations for alarm systems in WFDSs.

All participants expressed their opinion on the design appropriateness of the system based on the activation, deactivation, and notification of the alarm system, as shown in Figure 3. Of the 25 participants, the most influencing factor for activating an alarm was sound and vibration, followed by sound, vibration, and light, as seen in Figure 3. In alarm deactivation, the most important element is a physical button on the device. Finally, for the options of the alarm notification sent to the nominated relative or caregiver, most caregivers and older adults preferred to receive only a notification via text message or email, followed by the option of both a text message and phone call. Another question that researchers answered was: What measurement indicator would you employ to detect a fall? Their answers focused on the following measurements: acceleration, ground reaction force, and angular velocity. From these measurements, secondary indicators can be obtained such as the sum vector magnitude, displacements of the center of mass, angles, and angular momentum.


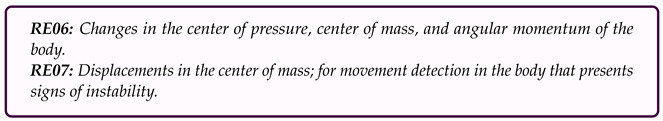


Some participants provided additional suggestions about the appropriateness of the systems. Researchers and health professionals recommended saving signal data collected from the system and involving family members or caregivers in assisting older adults when they request help. Other suggestions were that the device had a light source, and that it reported body decompensation, and that the system could be worn for other activities aside from fall detection.


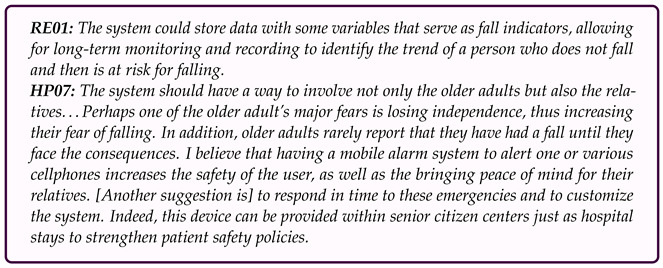


Additionally, researchers expressed their concerns and opinions about current issues, such as the reliability of devices due to false positives, and clinical validation.


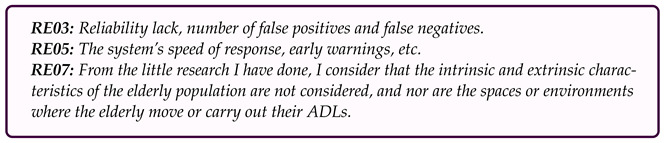


The informal caregivers and older adults for the most part said they would like to acquire the system:


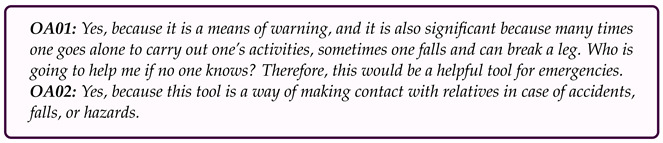


**Figure 4:**
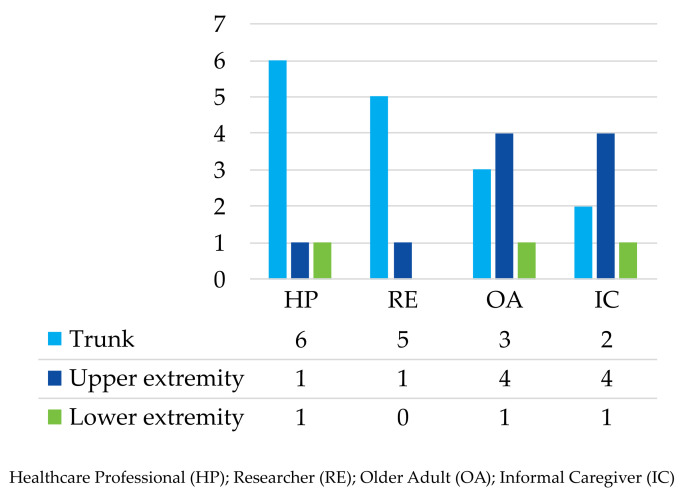
Summary of considerations for alarm systems in WFDSs.

For device location, this research divided responses into three, based on the parts of the body: the trunk, upper extremity, and lower extremity. The trunk was subdivided into the chest, waist, and hip, the upper extremity specified the wrist, and the lower extremity subdivided into the thigh and ankle. Of the 25 participants, 16 responded that the body trunk would be the best position to place the device, but ten would prefer the device to be worn on the upper extremity, as shown in Figure 4.

Most participants suggested a size for the WFDS, and most measurements were limited to the size of a smartwatch or smartphone. Specifically, physical appearance referred to size, weight, and usability. Some participants included not only the physical aspect but also the color, texture, and edges. Other participants expressed that the system could have distinguishable iconography and a harmonious presentation so that older adults are not “embarrassed” to use it.


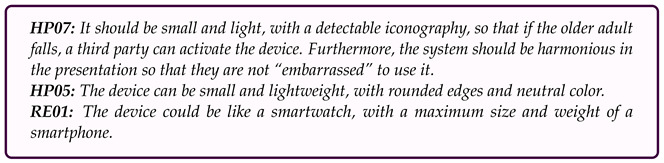


When asked if the older adult would like to use an WFDS, both informal caregivers and older adults responded in the affirmative. One reason given was the experience during a fall. For example, an older adult reported that he fell in their house, fell unconscious, and no one arrived to help them until he recovered consciousness. However, informal caregivers expressed obstacles such as the need to explain and justify the use of the system to a significant extent because older adults may feel that they are losing their independence.


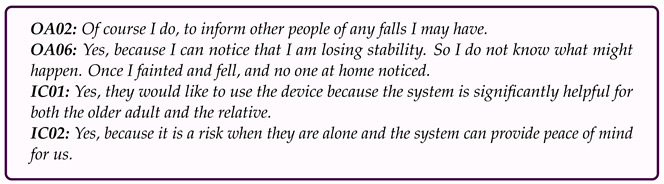


## 4. Discussion

### 4.1. Overview

While studies have been conducted on the perceptions of older adults using assistive technology [22,24,30] or fall detectors [23,29,31], the older adults consulted in Colombia reported having little knowledge of these devices. Recent information from a global report on assistive technology shows that there is a need for assistive technologies to enable people to live healthier, more productive, independent, and dignified lives, thus providing valuable support for those who wish to be more active in society. Such technologies can lead to socioeconomic benefit, such as decreased direct health and welfare costs, besides benefiting individuals and communities [16]. In addition, research concerning fall detectors found that there is a need to create systems with more accurate algorithms and technology that is functional yet comfortable [17,18,25,26]. The sample size used in this study, covering four stakeholder groups, offers a significant step forward in reporting the perception and knowledge of fall detectors in Colombia. A further contribution made by this study is in the recommendations made by the participants around fall detector development based on the requirements of some older adults in Colombia.

### 4.2. Falls and the Fall Detector Importance

Currently, the world’s population is aging, and the growing population of older adults wants to feel confident and capable in carrying out their ADLs and maintaining their independence, creating a strong link between caregiving for older adults and reducing complications from falls [12,13,20,27]. Likewise, assistive technologies have progressed over recent years; for example, fall detection is improving due to the constant advancements made in sensing elements, portability, comfort, accessibility, and accuracy [21]. In this study, the perceptions and recommendations received concerning fall detectors were determined by the needs of the stakeholders consulted, and the settings in which they would be used. For example, while the frequency of falls reported by participant older adults were few and mostly uneventful, older adults expressed their fears about future falls, such as that the next fall may be severe and require the assistance of a third party. They also reported that they required assistance for some ADLs, such as lifting heavy objects, reaching for things, or when they needed to climb stairs.

The main challenge in fall detection is differentiating between ADLs, near-falls, and falls [17,18,19,23]. These differences can be understood based on fall type, movements during falling, and recovery movements. In this study, the participants explained that the fall type that occurs most frequently is tripping and slipping. In addition, to avoid a fall, older adults develop compensatory movements to achieve stability. These movements are made with their upper or lower limbs, but subjects who do not develop compensatory movements may suffer severe injuries, such as hip fractures. Likewise, healthcare professionals expressed that physical exercise in the lower and upper limbs is important to strengthen muscle and build muscle mass, cardiovascular endurance, and balance, increasing competence in ADLs and increasing independence. In addition, the WHO reports that both genders suffer falls, but in some nations, men are more likely to die due to a fall, while women suffer non-fatal falls; in Colombia, this may be related to risk behaviors and specific hazards within the professions [6,7,8,11].

Although ADLs and falls can be identified by sensors placed on the body, some older adults expressed concern that they were being directly monitored, and wearing the system can be uncomfortable [17,25,26,27]. While there are differences in opinions among the participants on where to situate the device on the body, the majority expressed that the most suitable area is the trunk because the system has global data acquisition, providing information on postural stability and postural adaptations. Furthermore, the trunk is an uncomplicated location and a device would not interfere with the movements of upper limbs nor cause discomfort. Other participants suggested wearing a device on the wrist because it is a comfortable position to wear gadgets. However, in this location, system accuracy can be affected due to the range of motion typically made by the arms. They are commonly used in ADLs and such movements could be mistaken for a fall [17]. Another focus was the size of the fall detector. Most participants considered a proportion similar to or equal to a smartwatch (such as a 44 × 38 × 10.7 mm Apple Watch) because it is lightweight and portable. Research by Tanwar et al. [17] examines devices produced by different manufacturers, where the dimensions range from 66 × 38.1 × 15.2 mm (Medical Guardian) to 72.9 × 46.9 × 18.5 mm (Bay Alarm Medical), but the devices remain comfortable and safe.

Another challenge in fall detection is prompt attention by caregivers or relatives when a fall occurs. That is why participants provided views on how older adults would like to send an alert in the case of an emergency. Several participants considered an SMS or a phone call would be the best way to communicate with the elderly since telephones are accessible to over 50% of the Colombian population, and most older adults use this method of communication [15]. The device alarm is a key point in relation to fall detectors because the user may require help in different scenarios. For example, in the first scenario, a user falls and they need help, but the device does not activate; in the second scenario, the device presents a false alarm, and the user wishes to turn off the alarm; in these scenarios, the participants expressed that they would like to activate or deactivate the alarm with a button. Another scenario is when the device reports that the user has fallen and requires immediate help. For this scenario, the participants differed on their alarm ideas, as most proposed devices with sound and vibration or sound, light, and vibration. However, to develop multimodal fall detectors, the best option is to add the three peripherals because the vibration option helps older adults who have visual or hearing limitations, as well as the sound and lights that are visible to both the elderly and caregiver or relatives who are around or near the patient and can help them.

### 4.3. Existing and Approval of Fall Detectors

The results from this study show that a wide range of participants were not aware of the existence of fall detectors. However, some health professionals and researchers familiar with the devices responded that they were unreliable, as they produced significant false positives and false negatives. While false alarms seem to be a weak point in these devices, researchers are developing new strategies and sensors to reduce false positives, as shown in the studies conducted by [17,18,25,26,27]. Another disadvantage raised by a health professional is that this technology does not consider the specific fall risk factors of older adults (intrinsic and extrinsic), such as the spaces or environments where older adults perform their ADLs. Although the technology does not always consider the requirements of users, researchers are currently developing new devices that take into consideration stakeholder opinions [22,24,29,31]. Similarly, global entities such as the WHO and UNICEF have produced reports directed at government entities, global markets, users, developers, and investors, among others, to improve access to assistive technology, accrediting and encouraging the inclusion and engagement of people with disabilities and older adults [16,37].

The strong acceptance of this technology in this study may be due, in part, to the group sample since it is a small group of individuals who commonly use some type of technology, and most participants live in urban zones with access to communication and information technologies. In contrast, Munoz Garcia [15] revealed an existing gap between technology use and older adults in Colombia. Colombia’s national internet access by 2017 had reached 98.8% within affluent social groups, with less affluent social groups having a coverage of just 40%. There are significant disparities, particularly in access to and knowledge of technology in older adults; approximately 36.3% of older adults use the Internet, although almost 98% use cell phones [6,14,15].

Although some caregivers stated that the older adult in their care would be reluctant to adopt a device, they also expressed that older adults could manage it if they worked toward gaining greater competence in the use of technology, particularly if there was an understanding that this technology would improve their independence and safety. Considering these points, the world and regional health agencies and universities could conduct ongoing research on older adult healthcare, focusing on perception analysis and evaluating the fear, frustration, and nonacceptance of technological devices [22,29,31]. Yet, in Colombia, there are insufficient studies in these areas or on the acceptance of fall detectors by older adults; developments have focused on the technology but not on covering the necessities and expectations of the users.

### 4.4. Stakeholder Fall Detector Considerations

The main approaches in fall detectors have focused on instrument development and not on the convergence between the needs of older adults and the advancements of the technology [22,29,31]. In this study, the requirements of older adults are considered an essential part of WFDS development, concentrating on motivations, ideas, explanations, and factors influencing the use of WFDSs in daily life. The findings uncover that the system design involves the interaction between fall experiences, comfort, and safety.

The OA and IC participants suggest that the comfort of a system is far more important than the technological requirements. In particular, size and weight are essential factors because the final users consider that large systems would potentially be bothersome, embarrassing, or stigmatizing. Some stakeholders specified small device sizes and weights (around those of a smartwatch) so that devices can be hidden and practical, for example, worn as a pendant, around the waist, chest, or on the wrist. However, those in the HP and RE groups proposed that the best location for such devices is the trunk, positioned either at the body’s center of mass or center of gravity. This position can provide information about postural stability, it is not affected by limb movements, and may cause minimal discomfort when carrying out ADLs.

The findings around safety suggest that the WFDSs should meet the potential additional needs of the elderly, such as those associated with visual and hearing impairments, as well as sending alert messages with geolocation to people who can help them promptly. The suggestions made by stakeholders included the use of sound, vibration, light, and GPS. Furthermore, systems that can communicate with widespread hardware devices that they are comfortable using, such as smartphones that send messages or make calls for help. However, smartphone use may require an app with a user-friendly interface, appropriate for older adults, given the limited expertise of some older adults in smartphone use.

## 5. Conclusions

Assistive technology in this study refers to devices with particular characteristics and includes approaches to identify the type of assistive technology that enables people to feel safe by raising the alarm to increase the likelihood of receiving timely help, and supporting independent living. The methods of improving access to assistance tech products and redesigning devices must be oriented to more closely meet the needs of stakeholders.

This study investigated some cases of falls in Colombian older adults, such as how they were affected and assisted. Although WFDSs are available in countries such as Spain, as mentioned by a healthcare professional, in Colombia, knowledge or use of these systems is rare due to a deficiency of providers, the lack of training in them, and awareness-raising, as well as limited knowledge of national legislation and strategies that help the elderly receive timely care in the event of a fall or emergency. This study questioned stakeholders’ knowledge about FDSs and what factors could determine whether an FDS may be appropriate in meeting seniors’ needs. The participants mentioned the importance of these assistive products, although they emphasized their lack of knowledge and experience in the use of this assistive technology type for the safety of older adults. In addition, this study identified fall detectors as priority support products for Colombian older adults, as described by surveyed stakeholders. This research identified that assistive products increase older adults’ self-esteem, well-being, and motivation to perform ADLs. However, these products have some weaknesses, as described by some participants, who raised the issue that older adults feel anxiety and worry about wearing a device on their bodies continuously. This study contributes to the field of assistive technology by raising awareness of FDSs for the safety of older adults and providing information on designing FDSs according to the necessities of Colombian seniors. Its results are also helpful for analyzing the perceptions of participants around assistive technology access in Colombia.

## Figures and Tables

**Table 1:** Classification of main findings on WFDS perceptions and recommendations.

Section	Theme	Sub-Theme
Fall Characteristics	ADL	GARS
	Assessment	Frequency
		Method
		Rank
	Fear	Injuries
	Form	Fall movements
		Recovery movements
		Types
	Quantity	
	Research	Area
		Time
		Topic
Wearable Fall Detection Technology	Accessibility	Disadvantages
	Affordability	Acceptance
		Advantages
	Appropriateness	Additional suggestions
		Design
		Disadvantages
		Influencing factors
	Availability	Existing knowledge
	Usability	Advantages
		Comfort
		Influencing Factors

ADL = Activities of daily living; GARS = Groningen activity restriction scale.

## Data Availability

Not applicable.
